# Analysis of Flavonoids in Lotus (*Nelumbo nucifera*) Leaves and Their Antioxidant Activity Using Macroporous Resin Chromatography Coupled with LC-MS/MS and Antioxidant Biochemical Assays

**DOI:** 10.3390/molecules200610553

**Published:** 2015-06-08

**Authors:** Ming-Zhi Zhu, Wei Wu, Li-Li Jiao, Ping-Fang Yang, Ming-Quan Guo

**Affiliations:** 1 Key Laboratory of Plant Germplasm Enhancement and Specialty Agriculture, Wuhan Botanical Garden, Chinese Academy of Sciences, Wuhan 430074, China; E-Mails: mzzhucn@hotmail.com (M.-Z.Z.); yangpf@wbgcas.cn (P.-F.Y.); 2 Changchun University of Chinese Traditional Medicine, Changchun 130000, China; E-Mails: weiwutian@hotmail.com (W.W.); jiaoaj@hotmail.com (L.-L.J.)

**Keywords:** lotus leaves, antioxidant activity, macroporous resin chromatography, mass spectrometry

## Abstract

Lotus (*Nelumbo nucifera*) leaves, a traditional Chinese medicinal herb, are rich in flavonoids. In an effort to thoroughly analyze their flavonoid components, macroporous resin chromatography coupled with HPLC-MS/MS was employed to simultaneously enrich and identify flavonoids from lotus leaves. Flavonoids extracted from lotus leaves were selectively enriched in the macroporous resin column, eluted subsequently as fraction II, and successively subjected to analysis with the HPLC-MS/MS and bioactivity assays. Altogether, fourteen flavonoids were identified, four of which were identified from lotus leaves for the first time, including quercetin 3-*O*-rhamnopyranosyl-(1→2)-glucopyranoside, quercetin 3-*O*-arabinoside, diosmetin 7-*O*-hexose, and isorhamnetin 3-*O*-arabino- pyranosyl-(1→2)-glucopyranoside. Further bioactivity assays revealed that these flavonoids from lotus leaves possess strong antioxidant activity, and demonstrate very good potential to be explored as food supplements or even pharmaceutical products to improve human health.

## 1. Introduction

Lotus (*Nelumbo nucifera*), a common perennial aquatic herb, is extensively cultivated in eastern Asia, particularly in China [1]. All parts of lotus, including the leaves, stamens, flowers, seeds and rhizomes, have been used as traditional Chinese medicines or vegetables for thousands of years [2]. The leaves of lotus are traditionally used for the treatment of haematemesis, haematuria, metrorrhagia, hyperlipidaemia, fever and inflammatory skin conditions [3]. In recent years, the antioxidant [4], antiviral [5], anti-obesity [6] and lipolytic activities [7] of lotus leaves have been reported and attracted more and more interest, while phytochemicals from lotus leaves and their associated potential activities have not yet been fully explored. The annual production of lotus leaves now exceeds 800,000 tons in China, but most of them are discarded as agricultural wastes by farmers [8]. It is thus highly desirable to expedite the research on how to make the best use of lotus leaves as potential products for the food or pharmaceutical industries. In this regard, it is of primary importance to conduct a thorough analysis of the major chemical components from lotus leaves and their associated bioactivities.

It is reported that the lotus leaves are rich in flavonoids [5] and alkaloids [9], and several flavonoids have been isolated. Kashiwada *et al.* isolated and identified five flavonoid glycosides (quercetin 3*-O*-β-d-glucuronide, quercetin 3*-O*-β-d-xylopyranosyl-(1→2)-β-d-galactopyranoside, rutin, isoquecitrin and hyperin) by nuclear magnetic resonance spectroscopy (NMR) [5]. Ohkoshi *et al.* also identified eight flavonoids (quercetin 3-*O*-α-arabinopyranosyl-(1→2)-β-d-galactopyranoside, rutin, (+)-catechin, hyperoside, isoquercitrin, quercetin, and astragalin) [7]. Till now, ten flavonoids were isolated from lotus leaves [5,7,10]. Flavonoids, a type of phenolic compounds, have attracted extensive attention because of their strong antioxidant activity and their ability to reduce the formation of free radicals and to scavenge free radicals [11,12,13]. It is considered that oxidative damage is attributable to excess active oxygen species generated in the body [14], and the antioxidant defense system plays an important role in human health [15]. In order to reduce damage to the human body and prolong the storage stability of foods, synthetic antioxidants are often used in these processes [16]. However, the use of synthetic antioxidants, such as butylated hydroxyanisole (BHA) and butylated hydroxytoluene (BHT), has been questioned for the possibility of being carcinogenic and causing liver damage [17]. Compared with synthetic antioxidants, antioxidants from natural sources are characterized by their less toxicity, and better health effects. Thus, the focuses of this study were put on the flavonoids from lotus leaves and their associated antioxidant activity. 

So far, several methods for the enrichment and separation of flavonoids in lotus leaves including liquid-liquid extraction [10], SPE [18], high-speed counter-current chromatography (HSCCC) [19] have been developed. However, these methods have several limitations, such as low capacity, low yields, or the need for special instrumentation. Furthermore, these methods share similar difficulties in completely separating flavonoids and alkaloids, which are the major active ingredients of lotus leaves. Comparatively, macroporous resin chromatography, with its properties of high adsorption capacity, good stability, low operational cost, and simple procedure, is one of the most efficient methods to separate bioactive components from crude herbal material extracts [20]. Nowadays macroporous resins chromatography has been successfully applied in industry for separation and preparation of flavonoids, glycosides, saponins, and so on [21]. In this context, we strove to develop an off-line two-dimensional chromatography method combining macroporous resin and reverse phase liquid chromatography to firstly enrich the flavonoids from the crude extracts of lotus leaves in the first dimension, then conduct the subsequent chemical and activity analysis of the flavonoid components. 

## 2. Results and Discussion

### 2.1. Analysis of Flavonoids by HPLC-MS/MS

**Figure 1:**
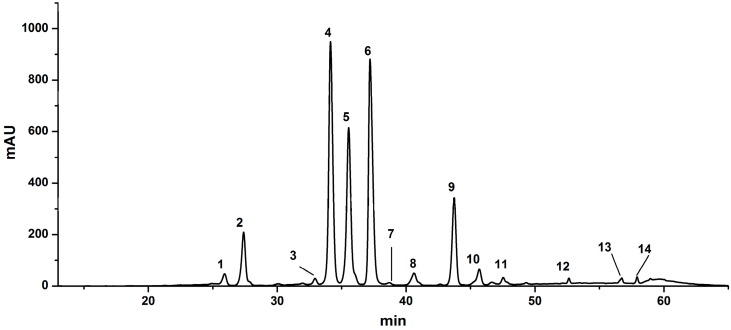
The HPLC profile of flavonoids in fraction II from lotus leaves recorded at 350 nm.

**Figure 2:**
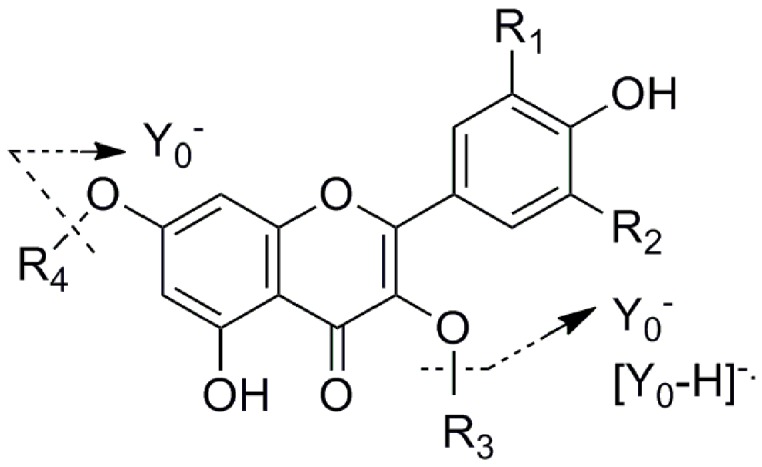
The MS/MS model of flavonoid *O-*glycosides.

The highest total flavonoid content (TFC) was determined in fraction II (690.5 mg isoquercetin equivalents/g sample, Table 1), while very few peaks were detected in fractions I, III and IV in chromatographic profiles recorded at 350 nm, so fractions I, III and IV were thus deemed to have very few or even no flavonoids. A successful enrichment and separation of total flavonoids from lotus leaves was thus achieved by the D101 macroporous resin chromatography, and the resultant flavonoids were then ready for the subsequent chemical and bioactivity analysis. After the flavonoids from the lotus leaves extracts were successfully enriched in fraction II using the D101 macroporous resin chromatography, HPLC-MS/MS was then employed to identify those flavonoids. Figure 1 shows the chromatographic profile of fraction II at 350 nm, where fourteen peaks were well detected and resolved, representing at least 14 lotus leaves flavonoids. To identify these already resolved flavonoids, LC-MS/MS experiments were conducted. The LC and MS/MS data, including retention times, molecular ions, aglycone ions and some important fragment ions are listed in Table 1. The fragmentation of *O-*glycosyl flavonoids in the negative ion mode (NI) is characterized by the loss of the sugar moieties, and deprotonated aglycone species (Y_0_^−^) or radical aglycone ion ([Y_0_− H]^−.^) fragments are obtained [22,23]. The MS/MS model of flavonoid *O-*glycosides is shown in Figure 2. For flavonol mono*-O*-glycosides, glycosylation took place at the 3-position if the relative abundance of the [Y_0_ − H]^−^^.^ ion was significantly higher than that of the Y_0_^−^ ion, and the situation is reversed when glycosylation happens at the 7-position [22]. Till now, five aglycones, kaempferol, quercetin, isorhamnetin, myricetin and diosmetin, were identified from lotus [24].

As listed in Table 1, peak 1 showed a [M − H]^−^ ion at *m/z* 479, with its [Y_0_− H]^−.^ ion at *m/z* 316 (loss of a hexose moiety), indicating that it was myricetin monohexoside, and the glycosylation took place at the 3-position based on the characteristic [Y_0_− H]^−.^ fragment ion. Peak 1 was therefore identified as myricetin 3*-O*-hexose, which has been previously found in lotus leaves and flowers [24]. Similar to the MS/MS model of peak 1, the presence of a [M − H]^−^ ion at *m/z* 477 and the corresponding [Y_0_ − H]^−.^ ion at *m/z* 314 for peak 13 indicated that it was an isorhamnetin monohexoside identified as isorhamnetin 3*-O*-hexose, which has been previously reported [18]. Peak 2 exhibited the [M − H]^−^ ion at *m/z* 595 with its [Y_0_ − H]^−.^ ion at *m/z* 300 (loss of a pentose and a hexose moiety), indicating that it was a quercetin diglycoside. For flavonol *O-*diglycosides, the mass spectrometric behaviors of diglycosides are notably different depending on the linkage between the two monosaccharides. A [Y_0_ − H]^−.^ ion tends to be generated in the case of a C1→C2 linkage between the two monosaccharides, while the Y_0_**^−^** ion is indicative of a C1→C6 linkage [22]. The higher abundance of [Y_0_− H]^−.^ ion at *m/z* 300 for peak 3 indicated that the interglycosidic linkage between the two monosaccharides in this compound was C1→C2, and peak 2 was thus identified as quercetin 3*-O*-arabinopyranosyl-(1→2)-galactopyranoside, which has been reported in lotus leaves [25]. 

**Table 1:** Identification of flavonoids in fraction II of lotus leaves by LC-MS/MS.

Peak No.	Rt (min) ^a^	NI-MS	MS/MS	Identification
1	25.9	479	316	Myricetin 3-*O*-hexose
2	27.4	595	300	Quercetin 3-*O*-arabinopyranosyl-(1→2)-galactopyranoside
3	33.0	609	300	Quercetin 3-*O*-rhamnopyranosyl-(1→2)-glucopyranoside
4	34.1	463	300	Quercetin 3-*O*-galactoside (hyperoside)
5	35.5	463	300	Quercetin 3-*O*-glucoside (isoquercitrin)
6	37.2	477	301	Quercetin 3-*O*-glucuronide
7	38.7	433	300	Quercetin 3-*O*-arabinoside
8	40.6	447	284	Kaempferol 3-*O*-galactoside
9	43.7	447	284	Kaempferol 3-*O*-glucoside (astragalin)
10	45.7	461	285	Kaempferol 3-*O*-glucuronide
11	47.5	461	446; 298; 283	Diosmetin 7-*O*-hexose
12	52.6	609	314; 299	Isorhamnetin 3-*O*-arabinopyranosyl-(1→2)-glucopyranoside
13	56.7	477	314	Isorhamnetin 3-*O*-hexose
14	57.9	491	315	Isorhamnetin 3-*O*-glucuronide

^a^ Rt: retention time on HPLC.

**Figure 3:**
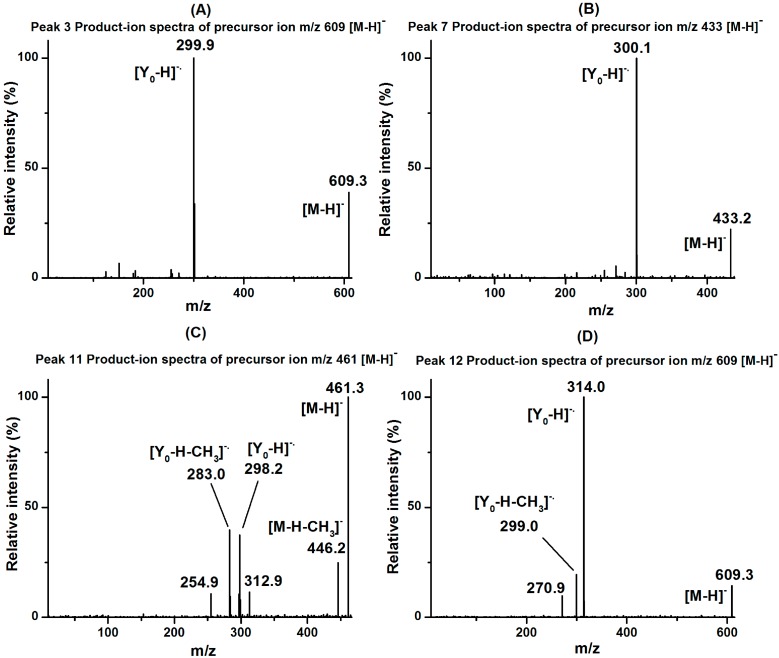
The MS/MS spectra of newly identified flavonoids in fraction II from lotus leaves: (**A**) peak 3; (**B**) peak 7; (**C**) peak 11; (**D**) peak 12.

As shown in Figure 3A,D, peaks 3 and 12 exhibited the same [M − H]^−^ ions at *m/z* 609 with [Y_0_ − H]^−^· ions at *m/z* 300 and 314, indicating that they were quercetin diglycoside and isorhamnetin diglycoside, respectively. Considering the similar MS/MS spectrometric behaviors with peak 2, peaks 3 and 12 were identified as quercetin 3*-O*-rhamnopyranosyl-(1→2)-glucopyranoside and isorhamnetin 3*-O*-arabinopyranosyl-(1→2)-glucopyranoside. These two flavonoids were identified in this study for the first time. In addition, the presence of the fragment ion at *m/z* 299 ([Y_0_ −H − CH_3_]^−^·) of peak 12 indicated that the methoxyflavone readily lost a methyl group [26].

Peaks 4 and 5 exhibited the same [M − H]^−^ ions at *m/z* 463 with [Y_0_− H]^−.^ ions at *m/z* 300 (loss of a hexose moiety), indicating that they are quercetin monohexoside isomers with a hexose conjugated at the 3-position. In addition, peak 4 had a shorter retention time than peak 5. Considering that glycosides linked with galactose elute before glucose linkages [27,28], peaks 4 and 5 were identified as quercetin 3*-O*-galactoside (hyperoside) and quercetin 3*-O*-glucoside (isoquercitrin) [25], and the results were further validated by comparing their LC-MS with the corresponding standards. 

Peak 6 showed a [M − H]^−^ ion at *m/z* 477, with a Y_0_^−^ ion at *m/z* 301 (loss of a glucuronic acid). For glucuronic acid glycosides, only the Y_0_**^−^** ion was observed during the MS/MS process, and the glucuronic acid glycoside was conjugated at the 3-position. Thus, peak 6 was identified as quercetin 3*-O*-glucuronide, which has previously been reported to be the major flavonoid in lotus leaves [25]. Like the MS/MS spectrometric behavior of peak 6, the ions at *m/z* 461 ([M − H]^−^), *m/z* 285 (Y_0_^−^), 491 ([M − H]^−^) and *m/z* 315 (Y_0_^−^) for peaks 10 and 14 indicated that they were kaempferol and isorhamnetin glucuronic acid glycosides, respectively. Thus, peaks 10 and 14 were identified as kaempferol 3*-O*-glucuronide and isorhamnetin 3*-O*-glucuronide [24,29].

As shown in Figure 3B, peak 7 showed a [M − H]^−^ ion at *m/z* 433, with a [Y_0_− H]^−.^ ion at *m/z* 300 (loss of a pentose moiety), indicating that it was quercetin monopentoside, and glycosylation took place at the 3-position. Peak 7 was therefore identified as quercetin 3*-O*-arabinoside. Its MS/MS spectra agreed with a compound identified in *Juglans regia* L. leaves [30], while it has been found in lotus leaves in this work for the first time. 

For peaks 8 and 9, the same [M − H]^−^ ions were observed at *m/z* 447, with their [Y_0_− H]^−.^ ions at *m/z* 284 (loss of a hexose moiety), indicated that both were kaempferol 3*-O*-hexoses. Furthermore, peak 8 had a shorter retention time than that of peak 9. Thus, peaks 8 and 9 were identified as kaempferol 3*-O*-galactoside and kaempferol 3-*O*-glucoside (astragalin), which have previously been reported in lotus petals [29]. In addition, peak 9 was further confirmed by comparing the LC-MS/MS spectra with the corresponding standard.

As shown in Figure 3C, peak 11 showed a [M − H]^−^ ion at *m/z* 461, with a [Y_0_− H]^−.^ ion at *m/z* 298 (loss of a hexose moiety), indicating that it was diosmetin monohexoside, and glycosylation took place at the 7-position. The presence of the ions at *m/z* 446 ([M – CH_3_]^−^) and 283 ([Y_0_ – H − CH_3_]^−.^) indicated that the aglycone of diosmetin easily lost a methyl group. Peak 11 was identified as diosmetin 7*-O*-hexose, which has also been found in lotus leaves for the first time.

### 2.2. Antioxidant Activity of Flavonoids from Lotus Leaves

The antioxidant activity of plant extracts cannot be evaluated by only one single method due to the complex nature of phytochemicals, and antioxidant activity determination is highly reaction-mechanism dependent [12]. Multiple chemical or biological assays have been developed to evaluate the antioxidant activity and explain the antioxidant mechanism of action of plant extracts. Of those, the DPPH assay, ABTS assay and reducing power assay are the most commonly used assays to evaluate the antioxidant activities of plant extracts [31]. In view of this, a series of assays including DPPH scavenging activity, ABTS scavenging activity and FRAP were used for the determination of the antioxidant activity of the fraction of lotus leaves. Results of these evaluations were expressed as Trolox equivalents and IC_50_ values, which are shown in Table 2. Fraction II showed good free radical scavenging activity in the DPPH assay. The activity of fraction II (4695.3 μmol·TE/g) was higher than that of BHT (3612.3 μmol·TE/g, positive control). In terms of IC_50_, fraction II (IC_50_ = 0.101 mg/mL) had a lower value than BHT (IC_50_ = 0.121 mg/mL), which implied that fraction II possessed a stronger radical scavenging activity in the DPPH assay. For scavenging activity pattern in the ABTS assay, the activity of fraction II (5012.3 μmol·TE/g) was higher than that of BHT (4567.0 μmol·TE/g), while the IC_50_ value of fraction II (IC_50_ = 0.138 mg/mL) was lower than that of BHT (IC_50_ = 0.143 mg/mL). With regard to the ferric reducing capacity of fraction II, the trend was almost the same as that of the DPPH and ABTS assay. The reducing power activity of fraction II (500.5 mmol Fe^2+^/100 g) was nearly the same with that of Trolox (642.1 mmol Fe^2+^/100 g).

**Table 2:** Total flavonoids in fraction II from lotus leaves ^a^ and their corresponding antioxidant activity.

Sample	Total Flavonoid (mg IE/g)	DPPH		ABTS		FRAP (mmol Fe^2+^/100 g)
		(μmol TE/g)	IC_50_ value ^b^ (mg/mL)	(μmol TE/g)	IC_50_ value ^b^ (mg/mL)	
Fraction II	690.5 ± 35.8	4695.3 ± 144.3	0.101 ± 0.007	5012.3 ± 133.8	0.138 ± 0.007	500.5 ± 62.8
BHT	nt	3612.3 ± 170.9	0.121 ± 0.004	4567.0 ± 155.6	0.143 ± 0.004	nt
Trolox	nt	nt	0.112 ± 0.005	nt	0.119 ± 0.005	642.1 ± 55.7

^a^ Each value is presented as the mean ± SD of three replicate determinations; ^b^ IC_50_ value was determined to be the effective concentration at which DPPH and ABTS radicals were inhibited by 50%, respectively. IE, isoquercetin equivalents; TE, Trolox equivalents; nt, not tested.

Taken together, the above results demonstrate that fraction II from lotus leaves possesses good antioxidant potential, and the antioxidant activity of fraction II was nearly the same as that of BHT. This capability may be correlated to the different flavonoid components identified in the lotus leaves, whose activity has been relative to the electron donating ability associated with the degree and position of hydroxylation and methoxylation on the B-ring [32]. The flavonoids possessing a catecholic B-ring, such as hyperoside (4) isoquercitrin (5) and quercetin 3*-O*-arabinoside (7) (which also possess a double bond at the 2 position of the C ring, conjugated with the 4-oxo group), are probably the derivatives that contribute the most to the total antioxidant activity, because the presence of a catechol moiety confers greater stability to the aroxyl radicals formed upon reaction with radical compounds. Kaempferol 3*-O*-galactoside (8) and astragalin (9), which only possess a double bond at the 2 position of the C ring (again conjugated with the 4-oxo function group) and a phenolic B-ring, probably contribute to the total antioxidant activity to a lesser extent than those of the compounds mentioned above.

## 3. Experimental Section

### 3.1. Chemicals and Materials 

Three flavonoid glycoside standards (quercetin 3*-O*-galactoside (hyperoside), quercetin 3*-O*-glucoside (isoquercitrin), kaempferol 3*-O*-glucoside (astragalin)) were purchased from Shanghai Tauto Biotech (Shanghai, China). HPLC-grade solvents (acetonitrile and formic acid), butylated hydroxytoluene (BHT), 1,3,5-tri(2-pyridyl)-2.4.6-triazine (TPTZ), 6-hydroxy-2,5,7,8-tetramethyl-chroman-2-carboxylic acid (Trolox), α,α-diphenyl-β-picrylhydrazyl (DPPH), and 2,2′-azinobis-(3-ethylbenzthiazoline-6-sulfonic acid) (ABTS) were purchased from Sigma-Aldrich Corp. (Shanghai, China). HPLC-grade water was obtained using a Milli-Q System (Millipore, Billerica, MA, USA). Other chemicals of analytical grade were obtained from Shanghai Chemical Reagent Corp. (Shanghai, China). Millipore membranes (0.22 μm) were purchased from Jinteng Experiment Equipment Corp. (Tianjin, China). D101 macroporous resin was purchased from an industrial chemical company affiliated with Nan Kai University (Tianjin, China). Fresh lotus leaves were collected from Guangchang (Jiangxi, China) in 2013 and stored at −40 °C. Lotus leaves were dried at 45 °C, and then stored at 4 °C before use.

### 3.2. Extraction and Fractionation of Crude Lotus Leaves Extracts

The dried lotus leaves were powdered (100 g) and ultrasonically extracted at room temperature for 40 min with 1000 mL of 70% ethanol. After three extractions, the extracts were combined and filtered, and the supernatants were evaporated under reduced pressure and lyophilized to afford dark-green residues which were dissolved in H_2_O (50 mL) and were subjected to liquid/liquid partitioning with petroleum ether (B.P. 60–90 °C) in order to remove chlorophyll. Later the lower layer was evaporated, and fractionated by the D101 macroporous resin chromatography. The column was washed with distilled water to remove water soluble impurities (sugar, protein, and other water-soluble molecules) and then eluted successively with 30, 50, 70, and 90% ethanol and named fractions I, II, III and IV, respectively. The weights of crude extracts and fractions (I–IV) were 27.6 g, 3.1 g, 3.7 g, 1.0 g and 1.1 g, respectively. Aliquots of the eluents were then subjected to further analysis.

### 3.3. Determinations of Total Flavonoids Content (TFC)

Total flavonoids content was determined using a previously described colorimetric method [33]. Briefly, a 30 μL aliquot of appropriately diluted sample solution was mixed with 180 μL of distilled water in a well of a 96-well plate and 10 μL of a 5% NaNO_2_ solution was added subsequently. After 6 min, 20 μL of 10% AlCl_3_ solution was added and allowed to stand for 6 min before an addition of 60 μL of 4% NaOH solution. The absorbance of the mixture was determined at 510 nm *vs.* a water blank using a multifunctional microplate reader (Infinite M200 PRO, Tecan, Männedorf, Switzerland) after 15 min. Isoquercitrin was used as standard compound for the quantification of total flavonoids. All values were expressed as milligrams of isoquercitrin equivalents per gram of sample (mg IE/g sample).

### 3.4. HPLC Analysis of Flavonoids

The analysis of flavonoids was carried out using a Thermo Accela 1250 U-HPLC system (Thermo Fisher Scientific, San Jose, CA, USA) equipped with a binary solvent pump, column oven, auto-sampler and UV detector. A 10-μL aliquot of each sample solution was injected and analyzed on a Sunfire C18 column (150 mm × 4.6 mm, 3.5 μm, Waters, MA, USA). The separation was conducted at 30 °C (column temperature) using a gradient elution method with 0.5% formic acid in distilled water (solvent A) and 0.1% formic acid in acetonitrile (solvent B). The solvent gradient in volumetric ratios was set as follows: 0–10 min at 88% A; 10–42 min from 88% A to 80% A; 42–55 min from 80% A to 70% A; 55–63 min from 70% A to 40% A; 63–64 min from 40 % A to 88% A; and 64–65 min at 88% A. The flow rate was 0.6 mL/min and the effluents were monitored at 350 nm.

### 3.5. Identification of Flavonoids 

Flavonoids were identified using a Thermo Accela 600 HPLC system with a UV detector coupled to a TSQ Quantum Access MAX triple-stage quadropole mass spectrometer (Thermo Fisher Scientific). Electrospray ionization (ESI) was applied in the negative ion mode (NI) for the MS analysis. The operation conditions of mass analysis were set as follows: capillary temperature, 350 °C; vaporizer temperature, 300 °C; sheath gas (N_2_) pressure, 40 arbitrary units; auxiliary gas (N_2_) pressure, 10 arbitrary units; spray voltage, 3 kV. The mass spectra were recorded in the mass range from *m/z* 150 to 1500. The MS/MS spectra were obtained using the Data-Dependent mode and the collision energy was set as following: collision energy (CE), 10 V; collision energy grad (CE grad), 0.035 V/m.

### 3.6. Determination of Antioxidant Activity of Flavonoids

#### 3.6.1. DPPH Free Radical Scavenging Activity

DPPH scavenging activity was determined by the method described by Brand-Williams *et al.* with slight modifications [34]. Ten μL of appropriately diluted sample or Trolox solution (31.25–1000 μM) was added to 190 μL of DPPH solution (final concentration was 0.1 mM in methanol) in a 96-well plate. Then, the sample mixture was shaken gently and kept in the dark at room temperature for 30 min. Thereafter, the absorbance at 517 nm was measured and methanol was used for the baseline correction with a multifunctional microplate reader. The DPPH radical scavenging activity of extracts was calculated from the standard curve of Trolox and expressed as micromoles of Trolox equivalents (TE) per gram of sample (μmol TE/g). In addition, to determine the IC_50_ of samples on DPPH, six different concentrations were used. Following the same procedure above, the methanol instead of sample was made as blank controls, while BHT and Trolox were used as positive controls. The ability to scavenge the DPPH radical was calculated as a percentage according to the following equation:

(1) DPPH-scavenging effect (%) = [(A_DPPH_ − A_S_)/A_DPPH_] × 100

where A_DPPH_ = absorbance of control, A_S_ = absorbance of sample), and the IC_50_ value was determined to be the effective concentration at which DPPH radicals were inhibited by 50%.

#### 3.6.2. ABTS Free Radical Scavenging Activity

ABTS free radical scavenging activity was determined according to the method adopted by Zou *et al.* with some minor modifications [33]. The ABTS assay is based on the capacity to quench ABTS radical cationic (ABTS^+^) formation relative to Trolox. Briefly, a solution of ABTS^+^ was prepared by mixing equal volumes of potassium persulfate (4.9 mM in H_2_O) and ABTS (7 mM in H_2_O), and the solution was incubated in the dark for 12–16 h. The radical was stable in this form for more than two days when stored in the dark at room temperature. The ABTS^+^ solution was then diluted with 80% ethanol to obtain an absorbance of 0.700 ± 0.005 at 734 nm. Afterwards, ten microliters of appropriately diluted samples was added to 190 μL of ABTS^+^ solution in a 96-well plate. The mixture was incubated in the dark at room temperature for 30 min and then the absorbance was recorded at 734 nm. Trolox was used as standard, and a standard calibration curve was obtained for Trolox at concentrations ranging from 31.25 μM to 500 μM. The ABTS free radical scavenging activity of samples was calculated from the standard curve of Trolox and expressed as micromoles of Trolox equivalents (TE) per gram of sample (μmol·TE/g). The scavenging activities of different concentrations of samples against ABTS^+^ were also measured to calculate the IC_50_, and the procedure was similar to the DPPH scavenging method described before.

#### 3.6.3. Ferric Reducing/Antioxidant Power (FRAP) Assay

FRAP assay was performed as described previously by Benzie and Strain [35]. This method measures the change in absorbance at 593 nm owing to the formation of a blue colored ferrous 2,4,6-tripyridyl-s-triazine complex (Fe^2+^-TPTZ) from colorless oxidized ferric form (Fe^3+^-TPTZ) by the action of electron donating antioxidants. The stock solutions included 300 mM acetate buffer, pH 3.6 (3.1 g C_2_H_3_NaO_2_·3H_2_O and 16 mL C_2_H_4_O_2_), 10 mM TPTZ solution in 40 mM HCl, and 20 mM FeCl_3_·6H2O solution. The working solution was prepared by mixing 10 volumes of acetate buffer, 1 volume of TPTZ solution, and 1 volume of FeCl_3_·6H_2_O solution and then warmed at 37 °C before use. Then, 10 μL of properly diluted samples and 30 μL of distilled water was added to 260 μL of freshly prepared FRAP reagent in a 96-well plate and mixed thoroughly. The mixture was incubated at 37 °C for 10 min, and the absorbance was measured at 593 nm. The FRAP value was calculated and expressed as millimoles of Fe^2+^ equivalents per 100g of sample (mmol Fe^2+^equiv/100 g) based on a calibration curve plotted using FeSO_4_·7H_2_O as standard at a concentration ranging from 0.125 to 2 mM. All solutions were freshly made and used on the day of preparation.

### 3.7. Statistical Analysis

Data were expressed as the mean ± standard deviation of triplicate measurements. The data were statistically analyzed using statistical software, OriginPro 8.6.

## 4. Conclusions

To extend our research on the correlations and underlying mechanisms between chemical components and their bioactivities, a more efficient macroporous resin chromatography with much better resolution and compatibility coupled with HPLC-MS was developed for the simultaneous separation and biochemical analysis of flavonoids from the complex extracts of lotus leaves. Altogether, fourteen flavonoids from lotus leaves were identified in this work, which greatly improved the previous LC-MS/MS method. More importantly, the newly developed method has led to some significant new findings. Among fourteen flavonoids identified, quercetin 3*-O*-rhamnopyranosyl-(1→2)-gluco- pyranoside, quercetin 3*-O*-arabinoside, diosmetin 7*-O*-hexose and isorhamnetin 3*-O*-arabino- pyranosyl-(1→2)-glucopyranoside were identified in lotus leaves for the first time. In addition, this macroporous resin chromatography method is very compatible with flexible downstream biological activity studies, and greatly facilitated the antioxidant activity screening in this study. It is expected that with some slight modifications this new method will prove useful to explore more important applications in food and pharmaceutical industries. 
